# Predictors for participation in DNA self-sampling of childhood cancer survivors in Switzerland

**DOI:** 10.1186/s12874-021-01428-1

**Published:** 2021-10-30

**Authors:** Nicolas Waespe, Sven Strebel, Denis Marino, Veneranda Mattiello, Fanny Muet, Tiago Nava, Christina Schindera, Fabien N. Belle, Luzius Mader, Adrian Spoerri, Claudia E. Kuehni, Marc Ansari

**Affiliations:** 1 Childhood Cancer Research Group, Institute of Social and Preventive Medicine, University of Bern, Bern, Switzerland; 2 CANSEARCH research platform for paediatric oncology and haematology, Faculty of Medicine, Department of Pediatrics, Gynecology, and Obstetrics, University of Geneva, Geneva, Switzerland; 3 Graduate School for Cellular and Biomedical Sciences (GCB), University of Bern, Bern, Switzerland; 4 Graduate School for Health Sciences (GHS), University of Bern, Bern, Switzerland; 5 Division of Pediatric Oncology and Hematology, Department of Women, Children, and Adolescents, University Hospital of Geneva, Rue Willy-Donzé 6, 1211 Genève, Switzerland; 6 Pediatric Oncology/Hematology, University Children’s Hospital Basel, University of Basel, Basel, Switzerland; 7 Center for Primary Care and Public Health (Unisanté), Institute of Social and Preventive Medicine (IUMSP), University of Lausanne, Lausanne, Switzerland; 8 SwissRDL - Medical Registries and Data Linkage, Institute of Social and Preventive Medicine, University of Bern, Bern, Switzerland; 9 Division of Pediatric Oncology and Hematology, Department of Pediatrics, University Hospital of Bern, Bern, Switzerland

**Keywords:** Childhood cancer, Cancer survivors, DNA, Cohort study, Drug side effects, Second primary neoplasm, Genetic predisposition, Registry, Genetic testing, Genetic polymorphism

## Abstract

**Background:**

Research on germline genetic variants relies on enough eligible participants which is difficult to achieve for rare diseases such as childhood cancer. With self-collection kits, participants can contribute genetic samples conveniently from their home. Demographic and clinical factors were identified previously that influenced participation in mailed self-collection. People with pre-existing heritable diagnoses might participate differently in germline DNA collection which might render sampling biased in this group. In this nationwide cross-sectional study, we analysed predictive factors of participation in DNA self-collection including heritable diagnoses.

**Methods:**

We identified childhood cancer survivors from the Swiss Childhood Cancer Registry for invitation to germline DNA self-sampling in September 2019. Participants received saliva sampling kits by postal mail at their home, were asked to fill them, sign an informed consent, and send them back by mail. Two reminders were sent to non-participants by mail. We compared demographic, clinical, and treatment information of participants with non-participants using univariable and multivariable logistic regression models.

**Results:**

We invited 928 childhood cancer survivors in Switzerland with a median age of 26.5 years (interquartile range 19-37), of which 463 (50%) participated. After the initial send out of the sampling kit, 291 (63%) had participated, while reminder letters led to 172 additional participants (37%). Foreign nationality (odds ratio [OR] 0.5; 95%-confidence interval [CI] 0.4-0.7), survivors aged 30-39 years at study versus other age groups (OR 0.5; CI 0.4-0.8), and survivors with a known cancer predisposition syndrome (OR 0.5; CI 0.3-1.0) were less likely to participate in germline DNA collection. Survivors with a second primary neoplasm (OR 1.9; CI 1.0-3.8) or those living in a French or Italian speaking region (OR 1.3; CI 1.0-1.8) tended to participate more.

**Conclusions:**

We showed that half of childhood cancer survivors participated in germline DNA self-sampling relying completely on mailing of sample kits. Written reminders increased the response by about one third. More targeted recruitment strategies may be advocated for people of foreign nationality, aged 30-39 years, and those with cancer predisposition syndromes. Perceptions of genetic research and potential barriers to participation of survivors need to be better understood.

**Trial registration:**

Biobank: https://directory.bbmri-eric.eu/#/collection/bbmri-eric:ID:CH_HopitauxUniversitairesGeneve:collection:CH_BaHOP Research project: Clinicaltrials.gov: NCT04702321.

**Supplementary Information:**

The online version contains supplementary material available at 10.1186/s12874-021-01428-1.

## Background

Cancer survivorship and associated health complications have become increasingly important with improved childhood cancer survival [1]. Chronic conditions like cardiac, pulmonary, auditory, endocrine, reproductive, and neurocognitive complications, and second primary neoplasms (SPNs) are gaining importance in research and clinical care [2]. By age 50 years, childhood cancer survivors have twice as many severe chronic health conditions compared to controls [3]. Mortality in survivors is more than 10-times higher than in the general population [4]. While many demographic, clinical, and treatment-related risk factors are known, the contribution of genetic variation in the development of health complications is still poorly understood [5–7].

Self-sampling of germline DNA by participants has increased the reach of sample collection particularly in target groups who do not attend regular medical care. Since the 1990’s buccal swabs are used. In the 2010’s saliva collection kits became widely available which are easy to use and yield DNA of good quality and sufficient quantity [8]. Saliva collection kits do not need time-sensitive processing or cooling like blood, but can be collected, transported, and stored in ambient conditions for years. Collection of saliva samples by participants themselves is feasible and effective [5]. Self-collection may, however, be affected by participation bias. Ness et al. identified female sex, white race/ethnicity, college graduation, never smoking, accessing the healthcare system in the past 2 years, and having a second malignant neoplasm as predictors for participating in saliva sample self-collection [5]. Outside of the US, predictors for participation in childhood cancer survivors have not been studied. Demographic and cultural differences might affect the perception of the healthcare system and acceptance of genetic research and lead to lower participation in some subgroups [9, 10]. Patients with heritable diagnoses underwent previous genetic workup and might therefore differ in their perception of genetic research compared to patients without known heritable diagnoses. Their willingness of participation in genetic research might differ which subsequently affects their inclusion in research. Heritable diagnoses have not been included in studies on DNA self-sampling, previously. We assessed the response rate over time, the influence of written reminders, and predictors for participation in DNA self-sampling in Switzerland as part of the national Germline DNA Biobanking for Childhood Cancer and Blood Disorders (BISKIDS) project.

## Methods

### Study design

For this cross-sectional study, we used contact information from the Swiss Childhood Cancer Registry (SCCR) at the Institute of Social and Preventive Medicine, University of Bern, Switzerland to invite childhood cancer survivors for germline DNA self-collection. Addresses were collected from hospitals involved in primary and follow-up care and updated through the Swiss national postal service.

We obtained data on patient characteristics from the SCCR. In September 2019, participants received self-sampling kits by postal mail at their home and were asked to send them to the germline DNA Biobank Switzerland for Childhood Cancer and Blood Disorders (BISKIDS), which is part of the Paediatric Biobank for Research in Haematology and Oncology (BaHOP) Geneva, Switzerland. All participants received an informed consent form together with the saliva kit. BaHOP was awarded the VITA label which certifies compliance of applicable ethical and legal frameworks and appropriate governance to biobanks by the Swiss Biobanking Platform (SBP; www.biobanksqan.ch/#/biobanks/3919). The SBP is the Swiss national coordination platform for human and non-human biobanks initiated by the Swiss National Science Foundation (https://swissbiobanking.ch). The Geneva Cantonal Commission for Research Ethics has approved the BaHOP biobank (approval PB_2017-00533) and the associated “Genetic Risks for Childhood Cancer Complications Switzerland (GECCOS)” study, which will utilize the samples (approval 2020-01723). We followed the recommendations of the STROBE statement to report the findings of our study (Supplementary Table 1).

### Study population

Eligible for invitation to our study were participants who were: (i) registered in SCCR; (ii) Swiss residents; (iii) treated in one of nine paediatric hospitals caring for children with cancers; (iv) diagnosed with a neoplasm according to the International Classification of Childhood Cancers, 3rd edition (ICCC-3), or Langerhans cell histiocytosis before age 21 years from 1976 to 2017; (v) exposed to lung toxic (chest radiotherapy) or ototoxic treatment (brain radiotherapy with ≥30 Gray or platinum chemotherapy); and (vi) survivors of 2 years or more since childhood cancer diagnosis as of July 2019 without upper age limit. We excluded participants who: (I) had declined participation in research projects; (II) had died; or (III) did not have a valid address in Switzerland.

### Outcome definition and clinical characteristics

Our main outcome was participation in the germline DNA sample collection, defined as returning the DNA sample and the signed consent form. Non-participation was defined as active decline or non-response until December 2020 (end of follow-up). Clinical information was extracted from the SCCR. We classified the age at first neoplasm in 5-year groups, calendar year at first neoplasm diagnosis and age at survey in 10-year groups, and the first neoplasm diagnoses into ICCC-3 main categories [11]. Chemotherapy, radiotherapy, and haematopoietic stem cell transplantation were classified as “yes” if given during the first neoplasm treatment. We classified cancer predisposition syndromes (CPSs) and second primary neoplasms (SPNs) as previously described [12]. In brief, we assigned underlying diseases as CPSs if they were reported in the current literature to be associated with an increased relative risk of neoplasms compared to the general population and SPNs according to the definitions of the International Agency for Research on Cancer.

### Sample collection

Survivors received a letter by postal mail with information on the planned DNA sample collection, and a form with which they could opt out and a prepaid return envelope. Those who did not opt out received 3 weeks later a parcel including (i) detailed information on the biobanking project and an informed consent form to sign, (ii) one Oragene DNA OG-500 saliva sampling kit (manufactured by DNA Genotek, Ottawa, Ontario, Canada) with material for return by regular mail (liquid-tight bio-specimen bag, bubble wrap), (iii) information on saliva sample collection and shipment provided by the manufacturer (www.dnagenotek.com/row/products/collection-human/oragene-dna/500-series/OG-500.html), (iv) a graphical abstract of the workflow of saliva collection and return of samples and consent forms, and (v) prepaid return envelopes for return of the consent form and the saliva sample. We sent two letters as reminders to those who did not return the sample, one after 6 weeks and another one after 8 weeks.

We offered participants the opportunity to receive additional information through (i) a project specific e-mail address, and (ii) a dedicated telephone hotline. Both were operated by the study coordinator, a trained specialist in paediatric haematology and oncology with experience in cancer genetics, or biobank staff in case of absence.

### Statistical analysis

We compared demographic, neoplasm, treatment, relapse, second neoplasm, and predisposition syndrome information of participants with non-participants. Univariable logistic regression and multivariable logistic regression models were fitted to identify determinants of participation. Covariates were kept in the multivariable logistic regression model using backward selection. We removed covariates with *p* ≥ 0.2. We additionally adjusted for sex and age at first primary neoplasm diagnosis in the model. We used the software Stata version 15 (Stata Corporation, Austin, Texas) for analyses. Statistical uncertainty of estimates was expressed as 95%-confidence intervals.

## Results

### Characteristics of cohort

**Fig. 1:**
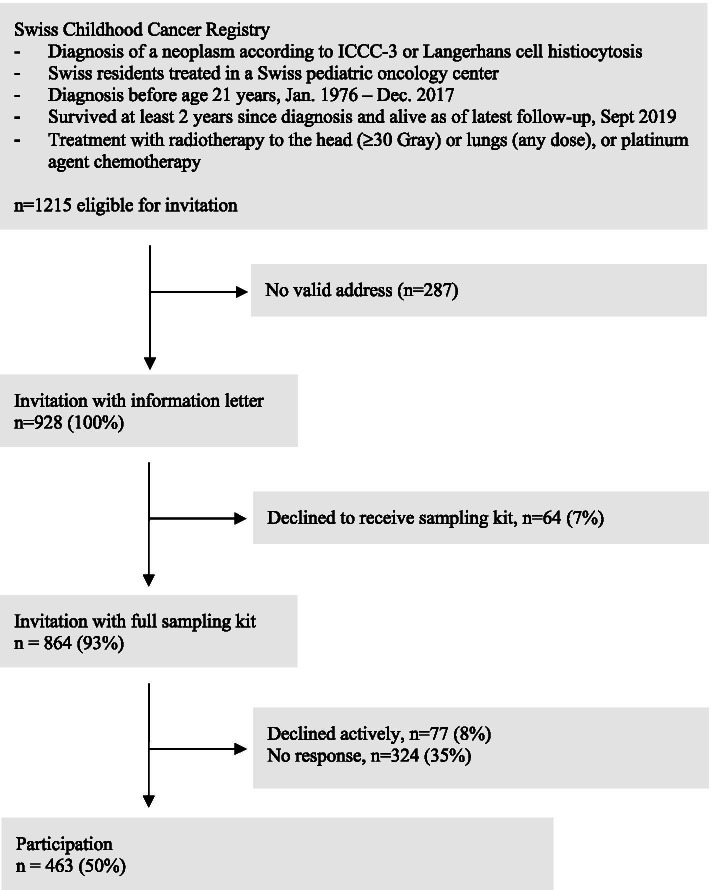
Flowchart of the response to invitation to the BISKIDS home collection of germline DNA in 928 cancer survivors. Legend: ICCC-3, international classification of childhood cancer, edition 3; n, number

We traced and contacted 928 of 1215 eligible childhood cancer survivors (Fig. 1). Of those we contacted, 463 (50%) returned a germline DNA sample. Median age at diagnosis was 8.7 years (interquartile range [IQR] 3-13) and at invitation 26.5 years (IQR 19-37; Table 1). The most common diagnoses were central nervous system tumours (28%) and lymphomas (20%) which reflected our selection process of inviting former childhood cancer patients with pulmotoxic and ototoxic treatments. Most patients had been treated with chemotherapy (93%) and radiotherapy (66%), while a minority underwent haematopoietic stem cell transplantation (7%). Relapse had been confirmed in 20% of survivors, a second primary neoplasm in 4% and a cancer predisposition syndrome (CPS) in 5%. Survivors were predominantly from the German (*n* = 633; 68%) language region and the remaining from the French (*n* = 262; 28%) and Italian (33; 4%) language region, which roughly reflects the language distribution in Switzerland.

**Table 1:** Characteristics of 928 Swiss childhood cancer survivors invited to home collection of germline DNA, stratified by participation status

	Participation			Univariable logistic regression		Multivariable logistic regression	
Characteristic	Total (n; %)^**a**^	Yes (n; %)^**a**^	No (n; %)^**a**^	OR (95%-CI)	***p***-value	OR (95%-CI)	*p*-value
**Sex**							
Female	426 (46)	209 (45)	217 (47)	Ref.		Ref.	
Male	502 (54)	254 (55)	248 (53)	1.06 (0.82 - 1.38)	0.64	1.09 (0.83-1.42)	0.53
**Nationality**							
Swiss	710 (76)	377 (81)	333 (72)	Ref.		Ref.	
Foreign	218 (24)	86 (19)	132 (28)	0.58 (0.42-0.78)	**< 0.001**	0.53 (0.38-0.73)	**< 0.001**
**Correspondence language**							
German	633 (68)	308 (67)	325 (70)	Ref.		Ref.	
French and Italian	295 (32)	155 (33)	140 (30)	1.17 (0.89-1.54)	0.27	1.31 (0.98-1.75)	0.07
**Age at first neoplasm diagnosis**							
Median (IQR; years)	8.7 (3.0-13.4)	8.7 (3.0-13.4)	8.6 (3.6-13.3)				
0-4 years	311 (33)	166 (36)	145 (31)	1.14 (0.83 – 1.57)	0.35	1.08 (0.75-1.56)	0.4
5-9 years	212 (23)	97 (21)	115 (25)	0.84 (0.59 - 1.20)		0.79 (0.54-1.14)	
10-14 years	287 (31)	144 (31)	143 (31)	Ref.		Ref.	
15-20 years	118 (13)	56 (12)	62 (13)	0.90 (0.58 - 1.38)		0.95 (0.61-1.48)	
**Year of diagnosis**							
1976-1985	110 (12)	54 (12)	56 (12)	0.84 (0.55 - 1.30)	**0.02**	‡	
1986-1995	193 (21)	78 (17)	115 (25)	0.59 (0.41 - 0.85)		‡	
1996-2005	312 (33)	164 (35)	148 (32)	0.97 (0.71 - 1.33)		‡	
2006-2016	313 (34)	167 (36)	146 (31)	Ref.		‡	
**Diagnostic group of first primary neoplasm (ICCC-3)**							
Leukaemias, myeloproliferative and myelodysplastic diseases	35 (4)	14 (3)	21 (5)	0.77 (0.37-1.58)	0.38		
Lymphomas	190 (20)	95 (21)	95 (20)	1.15 (0.79-1.68)			
CNS tumours and miscellaneous intracranial and intraspinal neoplasms	256 (28)	119 (26)	137 (29)	Ref.			
Neuroblastoma and other peripheral nervous cell tumours	78 (8)	45 (10)	33 (7)	1.57 (0.94-2.62)			
Retinoblastoma	44 (5)	18 (4)	26 (6)	0.8 (0.42-1.53)			
Renal tumours	33 (4)	20 (4)	13 (3)	1.77 (0.85-3.71)			
Hepatic tumours	24 (2)	15 (3)	9 (2)	1.92 (0.81-4.54)			
Malignant bone tumours	100 (11)	56 (12)	44 (9)	1.47 (0.92-2.33)			
Soft tissue and other extraosseous sarcomas	94 (10)	48 (10)	46 (10)	1.2 (0.75-1.93)			
Germ cell tumours, trophoblastic tumours, and neoplasms of gonads	64 (7)	29 (6)	35 (8)	0.95 (0.55-1.65)			
Other malignant epithelial neoplasms and malignant melanomas	6 (< 1)	2 (< 1)	4 (< 1)	0.58 (0.1-3.2)			
Langerhans cell histiocytosis	4 (< 1)	2 (< 1)	2 (< 1)	1.15 (0.16-8.3)			
**Chemotherapy**							
No	61 (7)	25 (5)	36 (8)	Ref.			
Yes	867 (93)	438 (95)	429 (92)	1.47 (0.87-2.49)	0.15		
- With platinum agents	491 (53)	259 (56)	232 (50)	1.27 (0.98-1.64)	0.07		
**Radiotherapy**							
No	319 (34)	160 (35)	159 (34)	Ref.			
Yes	609 (66)	303 (65)	306 (66)	0.98 (0.75-1.29)	0.91		
- With head radiotherapy (≥30 Gy)	343 (37)	166 (36)	177 (38)	0.91 (0.70-1.19)	0.49		
- With chest radiotherapy	328 (35)	156 (34)	172 (37)	0.87 (0.66-1.33)	0.29		
**Haematopoietic stem cell transplantation**							
No	864 (93)	428 (92)	436 (94)	Ref.			
Yes	64 (7)	35 (8)	29 (6)	1.23 (0.74-2.05)	0.43		
**Relapse of first neoplasm**							
No	738 (80)	368 (79)	370 (80)	Ref.			
Yes	190 (20)	95 (21)	95 (20)	1.01 (0.73-1.38)	0.97		
**Cancer predisposition syndrome**							
No	879 (95)	442 (95)	437 (94)	Ref.		Ref.	
Yes	49 (5)	21 (5)	28 (6)	0.74 (0.42-1.33)	0.31	0.54 (0.29-1.0)	**0.05**
**Second primary neoplasm**							
No	889 (96)	438 (95)	451 (97)	Ref.		Ref.	
Yes	39 (4)	25 (5)	14 (3)	1.84 (0.94-3.58)	0.07	1.92 (0.97-3.82)	0.06
**Age at study invitation**							
Median (IQR; years)	26.5 (18.8-36.5)	25.1 (18.3-35.2)	27.7 (19.5-37.3)				
< 10 years	50 (6)	23 (5)	27 (6)	0.72 (0.4-1.32)	**0.005**	0.75 (0.39-1.43)	**0.002**
10-19 years	215 (23)	121 (26)	94 (20)	1.09 (0.77-1.56)		1.17 (0.81-1.70)	
20-29 years	296 (32)	160 (35)	136 (29)	Ref.		Ref.	
30-39 years	198 (21)	78 (17)	120 (26)	0.55 (0.38-0.8)		0.54 (0.38-0.79)	
40 or more years	169 (18)	81 (17)	88 (19)	0.78 (0.54-1.14)		0.72 (0.48-1.07)	

Legend: ‡, omitted from multivariable regression model for collinearity with age at study invitation; ^a^, column percentages are indicated; *CNS* central nervous system, *ICCC-3* international classification of childhood cancer, edition 3; *IQR* interquartile range; n, number

### Recruitment and impact of reminders

**Fig. 2:**
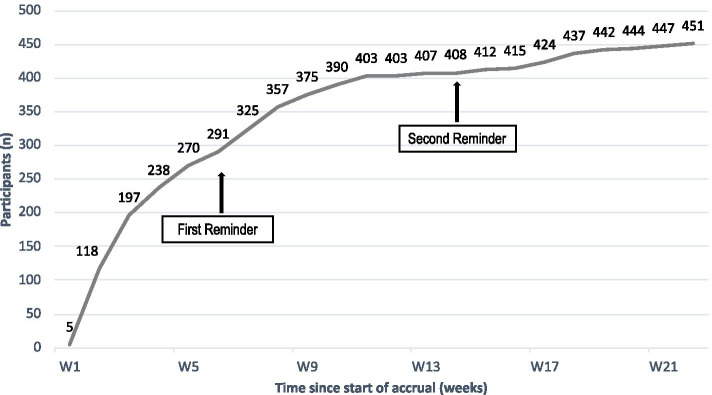
Recruitment over time of Swiss childhood cancer survivors invited for home germline DNA collection. Legend: n, number; W, week

We invited 928 survivors for home saliva collection. After the first information letter, 64 (7%) actively declined participation through an opt-out reply form. The remaining 864 survivors received a saliva sampling kit and an informed consent form. Seventy-seven of those actively refused participation (8%) and 324 eligible survivors did not answer (35%). Within 6 weeks after the send out of the sampling kit, 291 (63%) participated (Fig. 2). The first reminder letter led to 117 additional participants (25%) and the second reminder to an additional 55 participants (12%). On the consent form, 383 (83%) indicated that they wanted to be informed about potential incidental findings and 394 (85%) agreed that their samples could be used after death. Only a small proportion of invited people used the phone hotline (*n* = 32) or the e-mail contact provided (*n* = 31).

### Covariates associated with participation

We identified demographic and clinical covariates that differed between participants and non-participants. Foreign nationality (odds ratio [OR] 0.5; 95%-confidence interval [CI] 0.4-0.7) and age at study were associated with non-participation. Survivors aged 30-39 years at study invitation participated least (OR 0.5; CI 0.4-0.8), those 10-29 years tended to participate best, and those < 10 years and > 40 years in between. Those with a cancer predisposition syndrome tended to participate less (OR 0.5; CI 0.3-1.0), while survivors with a diagnosis of a second primary neoplasm (OR 1.9; CI 1.0-3.8) and those who spoke French or Italian (OR 1.3; CI 1.0-1.8) tended to participate more. There were no differences in sex, age at first primary neoplasm diagnosis, diagnostic groups, treatments, and relapse of first primary neoplasm between participants and non-participants.

## Discussion

This study described participation of Swiss childhood cancer survivors in home saliva collection for germline DNA extraction. We confirmed previous findings of about 50% participation in home DNA self-collection. Foreign nationality was a strong predictor for non-participation. Survivors with known CPSs and aged 30-39 years were also less likely to participate. In contrast, survivors with second primary neoplasms were more likely to participate. Most participating survivors wanted to be informed about incidental findings that might be relevant to their health and most agreed to their samples being available for research after their death.

The overall response rate of 50% was comparable to other DNA sample self-collections from home. Ness et al. reported 54% participation on 10,356 eligible adult childhood cancer survivors [5] and Dykema found 54% participation in 8081 adults in the Wisconsin Longitudinal Study [13]. Nishita et al. offered an incentive of 25US$ for returning a saliva sample which led to 59% participation of 967 adults in a smoking cessation trial, which was slightly higher than in studies without incentive [14]. Lower participation was found in genetic assessment of preterm birth where 23% of 708 mothers participated [15]. This study used an opt in approach where invited mothers were asked to reply by email, phone, text, or postal mail to indicate their interest in participating and then were sent DNA sampling kits. In contrast, we used a first letter explaining the study and giving the opportunity to indicate refusal of participation. All eligible people who did not opt out were then sent the sampling kit which might explain higher participation in our study. Even higher participation rates were achieved in an active clinical setting with patient-caregiver interaction where 97% of 155 adults from the US participated [16]. The close interaction of caregivers with participants and the possibility to address questions directly at invitation might have increased willingness to participate in the latter setting.

Foreign nationality was strongly associated with non-participation in concordance with a previous study from our group on response to questionnaire studies [17]. This could be related to language issues affecting understanding or reluctance to participate in research due to lower confidence in research. Foreign nationality is also linked to lower socioeconomic status, which was a predictor for lower participation in research [10]. Only few people used the provided phone or e-mail for further information. We did not differentiate the nationality in those who sought contact. Easy to understand information material, maybe in more languages, and invitation by phone might counteract these differences in participation. Our findings on language difference were in contrast to a large Swiss survey on willingness to participate in personalized health research [10], and our group’s previous publication on participation in a questionnaire study [17], where German speaking people were more likely to participate. The former was sent from the previously treating institutions and the latter from a German speaking research institution. In our study, the invitation was sent off from Geneva hospital, situated in the French speaking language region, which might have improved acceptance of the study of the people living in this language region. Survivors with known CPSs were less willing to participate in our study. Reasons could be the impression of futility as participants were already genetically investigated, fear of further findings, or bad experiences with genetic workup. Survivors with second primary neoplasms tended to participate more, potentially because they were seeking explanations for their multiple neoplasms. Lower participation in older survivors might be reflective of being more distanced to the previous disease or in a busy phase of their lives (caring for children and being active at work).

The approximate costs for preparation, sending out the samples, and for the send back by participants were similar to the price for the sampling kits themselves (around 20 Euros) and therefore doubled the costs per invited participant (around 40 Euros). The advantages of saliva samples are the ease of use (non-invasive collection), which is particularly relevant for paediatric populations, the reach of participants who do not attend regular medical care or who do not want to come to a hospital to participate in the study, and the possibility to store them at room temperature for years. While other germline DNA sampling techniques such as blood draws might be relatively inexpensive, the need for healthcare professionals to get the samples, quick processing or shipping, and cold storage, made saliva sampling kits cost-effective in previous studies [18].

More than 80% of participants wanted to be informed of incidental findings relevant to their health and agreed on further use of their samples after death. Our findings are in line with a previous study on the preference on information of incidental findings in genetic research [19]. A systematic review showed a high proportion of research participants who agreed on further use of genetic samples after the participant’s death [20]. There is still a minority who does not want to be informed of incidental findings or disagrees with use after death. In the setup of our biobank, we will account for these wishes.

A limitation of our study was that people who have died before study start could therefore not contribute DNA. We further did not collect information on reasons for non-participation. We also did not send additional sampling kits with reminders which might have prevented survivors from participating who had discarded their kit already. Strengths of the study were the availability of clinical information including CPSs. Our results of the study are likely representative for childhood cancer survivors in other countries with similar trust in healthcare institutions and responsible (genetic) research.

There are several areas that might be further investigated in future research to potentially improve participation. The optimal timing of reminders in mail-based study invitations has not been identified to our knowledge. We sent reminders out 6 weeks after the invitation and another 8 weeks after the first reminder, but shorter intervals could be tested. Phone reminders, a larger selection of languages, and possibly targeted invitation material for specific subgroups illustrating the importance of their contribution could also improve participation.

## Conclusion

Our study illustrates that home saliva collection through a process completely relying on mail is feasible and yields a response rate of about 50%. Specific survivor groups (foreign nationality, age 30-39 years, and patients with cancer predisposition syndromes) were less likely to participate which needs to be taken into account when studies are planned.

## Supplementary Information

**Additional file 1: Supplementary Table 1**. STROBE Statement for Swiss childhood cancer survivors invited for home germline DNA collection.

## Data Availability

The datasets that will be generated in subsequent studies using the collected materials will be available from the corresponding author on reasonable request through the clinicaltrials.gov identifier NCT04702321 or www.biobanksqan.ch/#/biobanks/3919.

## Abbreviations

BaHOP: Biobank for Haematology and Oncology in Paediatrics
BISKIDS: Germline DNA Biobank Switzerland for Childhood Cancer and Blood Disorders
CCS: Childhood cancer survivor
CNS: Central nervous system
CPS: Cancer predisposition syndrome
DNA: Deoxyribonucleic acid
GECCOS: Genetic risks for childhood cancer complications Switzerland
HSCT: Haematopoietic stem cell transplantation
ICCC-3: International Classification of Childhood Cancer, third edition
ISPM: Institute of Social and Preventive Medicine
SCCR: Swiss Childhood Cancer Registry
SPN: Second primary neoplasm
